# MultiTax-human: an extensive and high-resolution human-related full-length 16S rRNA reference database and taxonomy

**DOI:** 10.1128/spectrum.01312-24

**Published:** 2025-01-16

**Authors:** Zhiwei Bao, Bin Zhang, Jianhua Yao, Ming D. Li

**Affiliations:** 1 State Key Laboratory for Diagnosis and Treatment of Infectious Diseases, National Clinical Research Center for Infectious Diseases, National Medical Center for Infectious Diseases, Collaborative Innovation Center for Diagnosis and Treatment of Infectious Diseases, The First Affiliated Hospital, Zhejiang University School of Medicine, Hangzhou, China; 2 Joint Institute of Tobacco and Health, Kunming, Yunnan, China; Brown University, Providence, Rhode Island, USA

**Keywords:** human microbiome, 16S rRNA, GTDB, taxonomy, reference database

## Abstract

Considering that the human microbiota plays a critical role in health and disease, an accurate and high-resolution taxonomic classification is thus essential for meaningful microbiome analysis. In this study, we developed an automatic system, named MultiTax pipeline, for generating *de novo* taxonomy from full-length 16S rRNA sequences using the Genome Taxonomy Database and other existing reference databases. We first constructed the MultiTax-human database, a high-resolution resource specifically designed for human microbiome research and clinical applications. The database includes 842,649 high-quality full-length 16S rRNA sequences, extracted from multiple public repositories and human-related studies, offering a comprehensive and accurate portrayal of the human microbiome. To validate the MultiTax-human database, we profiled the human microbiome across various body sites, identified core microbial taxa, and tested its performance using an independent data set. Additionally, the database is equipped with a user-friendly web interface for easy querying and data exploration. The MultiTax-human database is poised to serve as a valuable tool for researchers, enhancing the precision of human microbiome studies and advancing our understanding of its impact on human health and diseases.

**IMPORTANCE:**

Understanding the human microbiome, the collection of microorganisms in and on our bodies, is essential for advancing health research. Current methods often lack precision and consistency, hindering our ability to study these microorganisms effectively. Our study presents the MultiTax-human database, a high-resolution reference tool specifically designed for human microbiome research. By integrating data from multiple sources and employing advanced classification techniques, this database offers an accurate and detailed map of the human microbiome. This resource enhances the ability of researchers and clinicians to explore the roles of microorganisms in health and disease, potentially leading to improved diagnostics, treatments, and insights into various medical conditions.

## INTRODUCTION

The human microbiota, an intricate community of microorganisms residing in and on the human body, has been increasingly recognized as a major player in human health and diseases. This complex microbial ecosystem is involved in numerous physiological processes, including digestion, immune function, and even behavior modulation (1–4). The recent advances of high-throughput sequencing technologies have revolutionized our ability to study these communities at breadth and depth previously unattainable (5).

While 16S rRNA sequencing has been instrumental in advancing microbiome research, the accuracy of taxonomic assignments still relies heavily on the quality of reference databases. High-resolution taxonomic classification is crucial for accurately identifying the composition and function of microbial communities, as well as for linking specific taxa to health outcomes or disease states. Despite the great progress has been made by current 16S rRNA databases and computational pipelines, many existing resources are limited by low taxonomic resolution (6, 7). This could result in ambiguous or incorrect assignments, particularly at the species or strain level. Additionally, inconsistencies between different databases and classification methods often lead to conflicting results, complicating the comparison and integration of microbiome data sets (8).

Recognizing these limitations, this study aimed to introduce the MultiTax-human database and the accompanying MultiTax pipeline with the following two objectives. The first objective was to enhance the resolution of taxonomic classification in human microbiome research, and the second one was to provide a consistent framework for the annotation and interpretation of 16S rRNA sequence data. By integrating full-length 16S rRNA sequences from a multitude of public databases, including Greengenes2 (9), SILVA (10), RDP (11), Genome Taxonomy Database (GTDB) (12), and reported human microbiome studies generated with different sequencing platforms such as Illumina (13, 14), PacBio (5), or Nanopore (15), our newly developed MultiTax-human in this study is expect to offer an extensive and detailed representation of the human microbiome, with an unprecedented level of details. It is our hope to set a new standard for microbiome research by offering the scientific community a robust platform for exploring the microbial frontier with greater accuracy and reliability.

## MATERIALS AND METHODS

### Data sets

The databases of Greengenes2 (9), SILVA (release 138.1) (10), RDP (11), and GTDB (release 207) (12) were all downloaded from their official websites. We procured human-associated 16S rRNA raw data from the NCBI Sequence Read Archive database. A search with the keywords of “Full-length 16S” AND “human” yielded a total of 1,798 fastq original sequence data files. We also included the sequencing data from the H120 data set, which contained 155 human fecal samples obtained from seven geographic regions (i.e., northeast, northwest, central, east, southeast, southwest, and south) across China, all of which were sequenced using the Pacific Biosciences (PacBio) Single Molecule Real-Time (SMRT) platform (16).

### Integration of public databases and reference database construction with MultiTax pipeline

The MultiTax automatic annotation pipeline is a modular multi-step Linux BASH script, which operates in three primary steps. First, it aligns high-quality full-length 16S rRNA sequences (full-length operational taxonomic units [OTUs]) in the same orientation relative to the reference database (GTDB) using the -orient parameter of usearch. This step ensures that all sequences are oriented in the 5′ to 3′ direction for uniformity and accuracy. The second step involves identifying the sequence’s closest match in GTDB to determine the percentage identity of the best hit, which was realized by using the -usearch_global command. The third step involves the classification of sequences. The MultiTax pipeline utilizes the GTDB classification as its backbone. It adheres to statistically supported identity thresholds at each taxonomic level, i.e., 75.0% for domain, 78.5% for phylum, 82.0% for order, 86.5% for family, 94.5% for genus, and 98.7% for species (17).

### Bioinformatics and biostatistics

For the full-length 16S rRNA sequences obtained from public 16S rRNA databases, we first performed quality control on them with our laboratory-developed Python script where sequences shorter than 1,200 base pairs were excluded, and sequences containing eight or more homopolymers or five ambiguous bases were discarded. The quality-controlled RDP, Silva, and Greengenes2 databases were then input into the MultiTax pipeline for re-annotation based on GTDB classification.

In parallel, the data set downloaded from NCBI underwent preliminary processing through fastp (18), an ultra-fast all-in-one FASTQ preprocessor. We employed the parameter of “--length_required 1000” to filter out any sequence <1,000 base pairs, leaving only high-quality and long sequences for further analysis. All other parameters were kept as default settings to maintain the generalizability of the data. For the post quality control, we performed zero-radius ZOTU clustering with usearch (v. 11) (19), which appears to be more stringent and accurate than traditional OTU clustering methods. The scripts used for data pre-processing are accessible on GitHub (https://github.com/zwbao/MultiTax-database). For downstream data analysis, we utilized the phyloseq (20) and microbiome R packages to estimate alpha diversity (species richness within a sample) and beta diversity (species diversity between samples).

In an effort to democratize access to our resources, we have developed a user-friendly interface: the MultiTax-human webpage, constructed utilizing the Shiny R package. This interactive platform empowers users to submit their query sequences and, in return, receive detailed classification information alongside insights into the sequences’ prevalence within various human body sites, in accordance with data derived from the MultiTax-human database.

## RESULTS

### Construction of MultiTax pipeline and MultiTax-human databases

**Fig 1:**
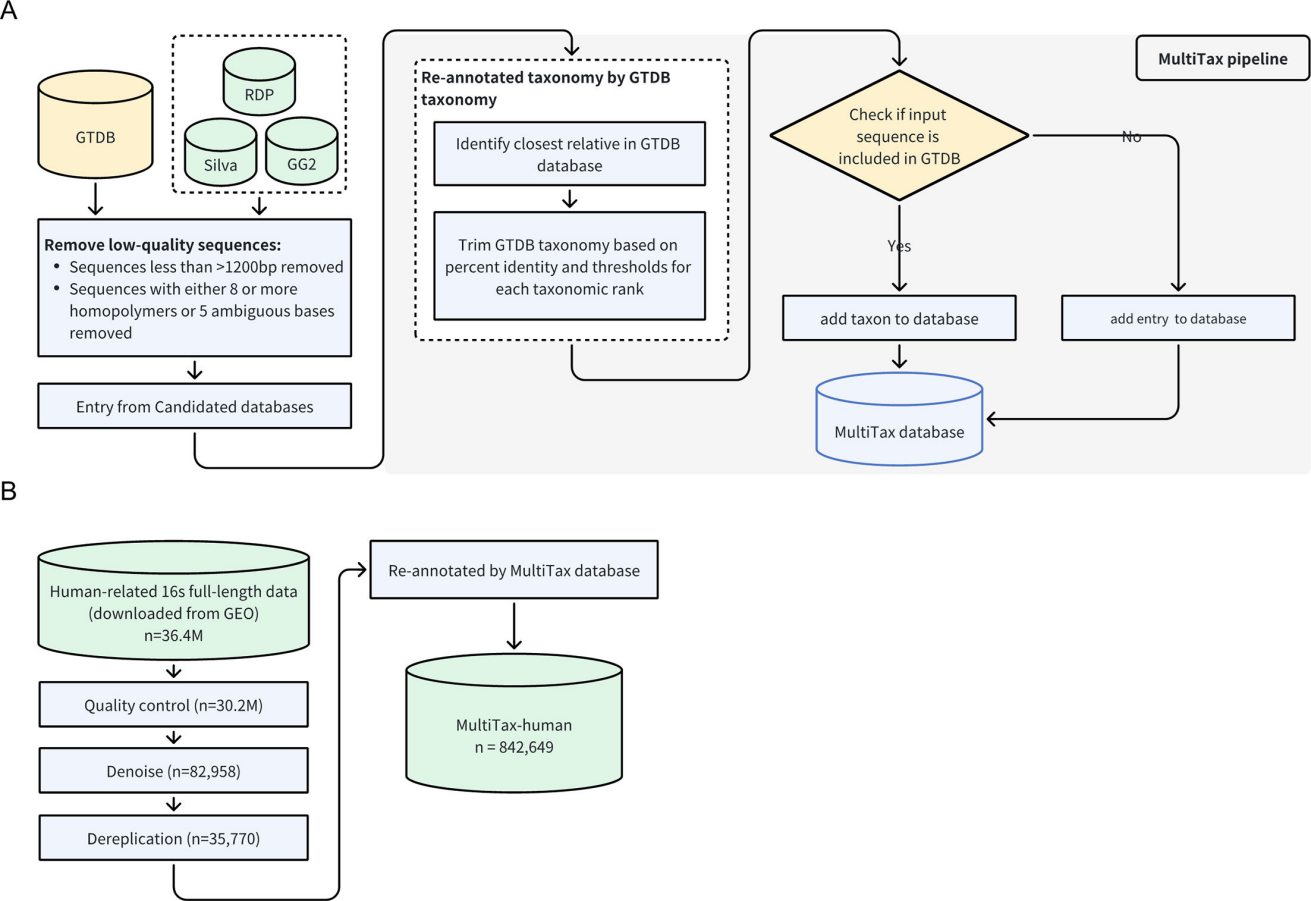
Construction of MultiTax reference and MultiTax-human databases. (**A**) The pipeline for constructing the MultiTax reference database. We applied quality control procedures to four widely used 16S rRNA databases: GTDB, Greengenes, Silva, and RDP, aiming to filter out low-quality 16S sequences. Sequences were removed if they were shorter than 1,200 base pairs or contained homopolymer stretches or ambiguous bases. The remaining sequences were re-annotated using a global alignment algorithm based on GTDB taxonomy, assigning names based on sequence identity and taxonomic thresholds. For sequences already present in GTDB (100% identity), annotations from other databases were integrated. For sequences not present in GTDB, we predicted a GTDB name and added these sequences to the MultiTax database. (**B**) The process for constructing the MultiTax-human database. We downloaded human-related full-length 16S rRNA data (36.4 million reads) from GEO. After applying quality control, denoising, and dereplication, 35,770 unique sequences remained. These were re-annotated using the MultiTax database, resulting in a final data set of 842,649 high-quality sequences in the MultiTax-human database.

The initial phase of this study involved the construction of both the MultiTax and MultiTax-human databases (Fig. 1). A stringent quality control process was deployed to obtain high-quality full-length 16S rRNA sequences from four widely acknowledged 16S rRNA databases, namely, GTDB, Greengenes, Silva, and RDP. We subsequently re-annotated the residual sequences from Greengenes, Silva, and RDP based on GTDB using a global alignment algorithm. This was accomplished by identifying the most comparable sequences in GTDB and assigning a GTDB name, which was influenced by sequence identity and taxonomic thresholds proposed by Yarza et al. (17). Sequences presented in GTDB (with 100% sequence identity) were incorporated into the MultiTax database with annotations from the other databases. For sequences not present in GTDB, we predicted a GTDB name, assigned annotations from other databases, and included the sequence and annotation information in the MultiTax reference database.

Additionally, we included preprocessed 1,798 human-related 16S rRNA full-length sequencing samples from NCBI. These samples underwent quality control, denoising, and deduplication and were merged with the MultiTax database using the same annotation method as GTDB. This amalgamation resulted in the MultiTax-human database, encompassing 842,649 high-quality full-length 16S rRNA sequences.

### Profiling the human-related microbiome using MultiTax-human database

**Fig 2:**
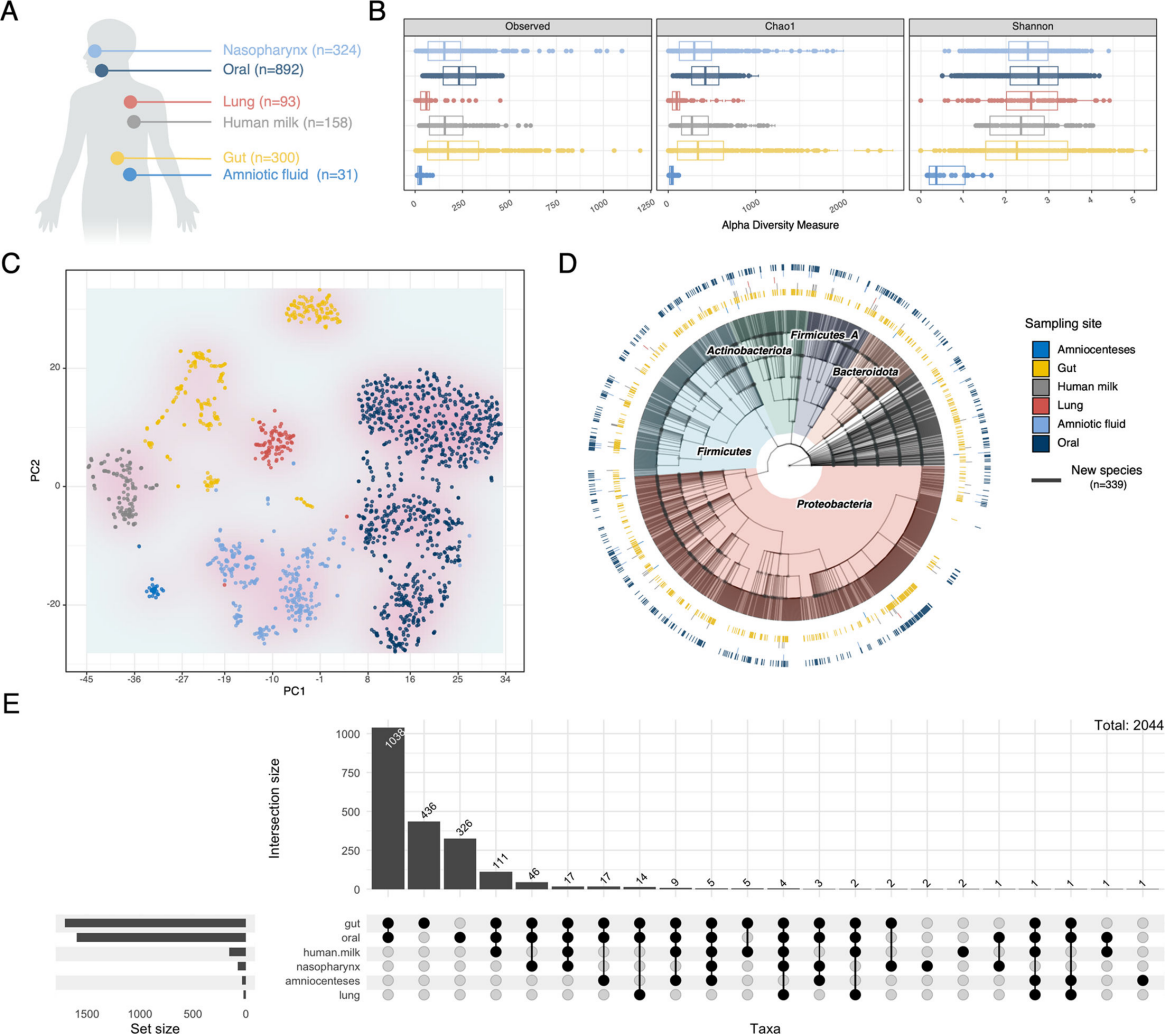
Profiling the human microbiome using MultiTax-human database. (**A**) A schematic representation of human sample sources. Our study samples include 324 nasopharyngeal samples, 892 oral samples, 93 lung samples, 158 human milk samples, 300 intestinal samples, and 31 amniocentesis samples. (**B**) A comparison of microbial alpha diversity from human body. (**C**) T-SNE representation of human microbiota patterns after Hellinger transformation and Euclidean distance estimation. (**D**) Phylogenetic tree of novel taxa annotated by MultiTax-human database. Novel species are highlighted by thick branches. The outer circle of the tree denotes the presence of each species in different sites with various colors. (**E**) Upset plot for assessing microbiota intersection across human body sites.

With the MultiTax-human database, it enabled us to do a thorough profiling of the human microbiome (Fig. 2). The data set downloaded from NCBI included 324 nasopharyngeal samples, 892 oral samples, 93 lung samples, 158 human milk samples, 300 intestinal samples, and 31 amniocentesis samples (Fig. 2A; Table S1). After performing a comparison of microbial alpha diversity across various human body samples (Fig. 2B), we found that the human oral microbiome exhibited the highest alpha diversity, whereas the amniotic fluid had the lowest. By employing Hellinger transformation and Euclidean distance estimation, we generated a t-SNE representation of human microbiota patterns (Fig. 2C). Phylogenetic analysis of potential new taxa in the MultiTax-human database revealed 339 new species, which were emphasized with bold branches (Fig. 2D). These newly discovered taxa were predominantly distributed in *Proteobacteria*, *Firmicutes*, *Actinobacteria*, *Firmicutes_A*, and *Bacteroidota* and mainly derived from human gut and oral samples. Additionally, we investigated the microbiota intersections across different human body sites (Fig. 2E), revealing a significant intersection between oral and gut microbiomes.

### Identification and analysis of human core microbial groups

**Fig 3:**
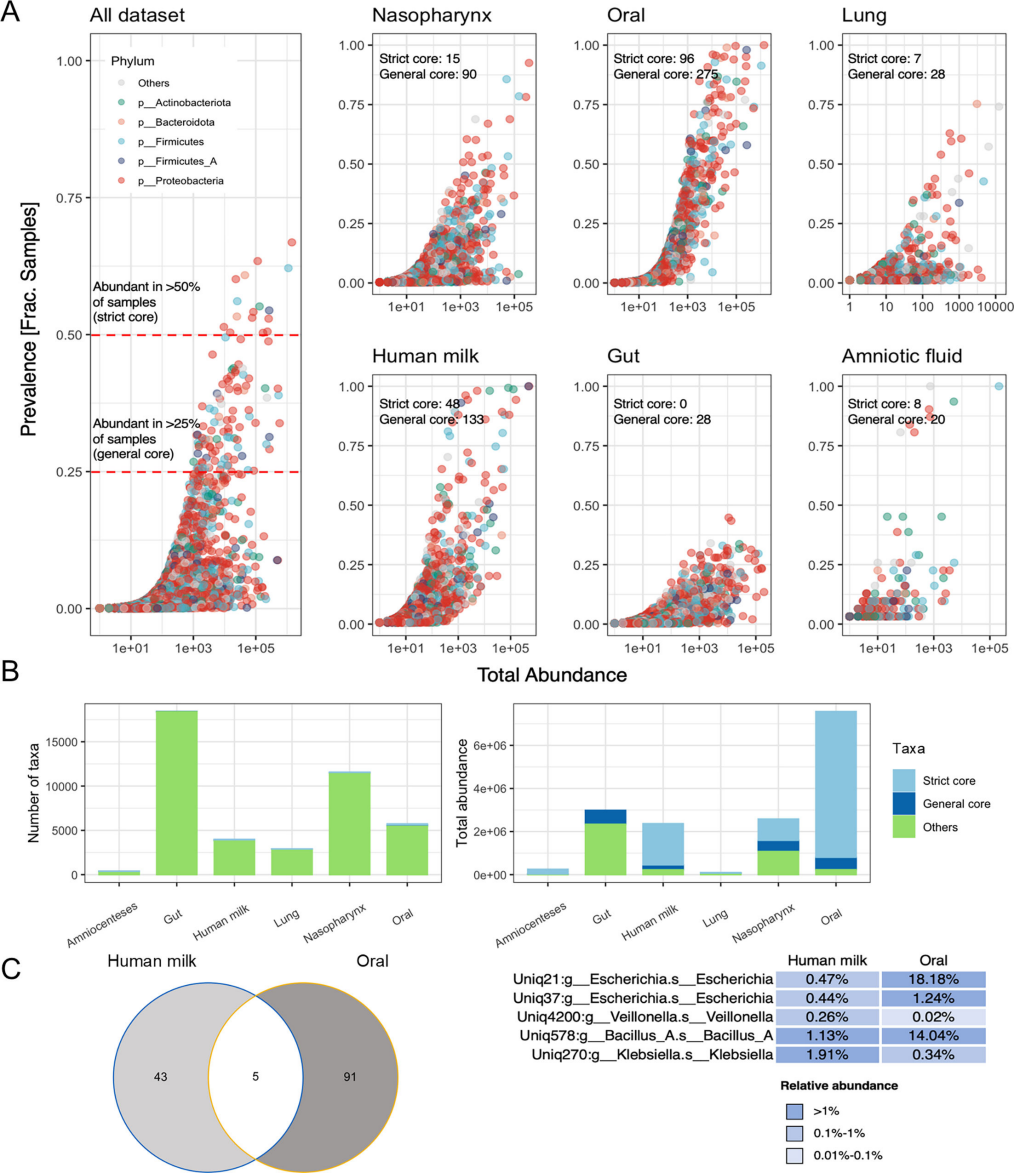
Identification and analysis of human core microbial groups in MultiTax-human database. We defined core microbiota as OTUs that were presented in more than 25% of the samples from a given body site and classified them into strict core (presented in >50% of the samples) and general core (presented in 25%–50% of the samples). (**A**) Scatter plots of human-derived microbes by body site. The left plot is a scatter plot of all human-derived microbes, where each dot represents an OTU, the x-axis is the total abundance of the OTU in all human samples, the y-axis is the occurrence frequency of the OTU in all human samples, and the color indicates the phylum of the OTU. The right plot is composed of six subplots, each showing the scatter plot of OTUs in one of the six body sites. (**B**) Composition of core and non-core microbiota by body site and abundance. (**C**) Comparison of core microbiota between human milk and oral samples.

Following the development of MultiTax-human database, we analyzed human core microbial groups within it (Fig. 3). We defined core microbiota as OTUs presented in over 25% of samples from a specific body site and further classified them into strict core (presented in >50% of the samples) and general core (presented in 25%–50% of the samples) (Fig. 3A). Subsequently, we compared the proportion of these core microorganisms in the total taxonomy and their abundance in the total abundance. The results revealed that despite their low proportion, these core microorganisms accounted for a significant portion of the total abundance, especially in amniotic fluid, breast milk, lungs, nasopharynx, and oral cavity (Fig. 3B). Additionally, we compared the core microbiota in breast milk and oral samples (Fig. 3C), highlighting a correlation between the two, with five OTU intersections in the strict core microbiota of human milk and oral.

### Evaluation of MultiTax-human database using H120 cohort

**Fig 4:**
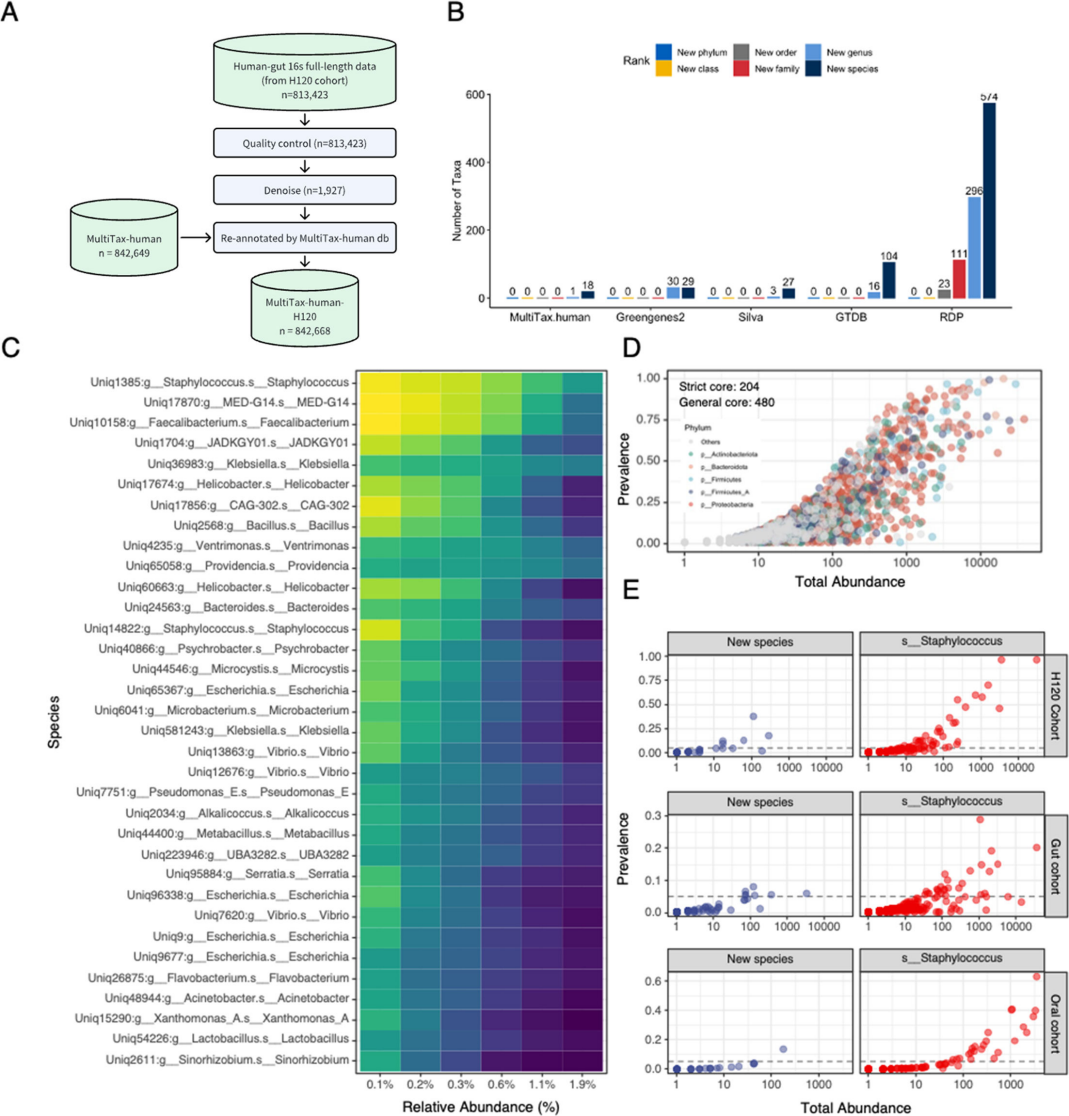
Evaluation of MultiTax-human database using H120 cohort. (**A**) Schematic diagram of the analysis workflow. The H120 data set comprises PacBio 16S full-length sequencing data from 122 Chinese healthy gut samples. The data were subjected to quality control and denoising and then assigned to taxa using MultiTax-human database. We detected 19 novel taxa that were absent in the original MultiTax database. (**B**) Comparison of the number of unclassified sequences by different databases at various taxonomic ranks. The H120 data set was annotated using MultiTax-human, GTDB, Greengenes2, Silva, and RDP databases. The y-axis shows the number of unclassified sequences, and the different colors indicate different taxonomic ranks. (**C**) Prevalence of core microbiota in H120 cohort at different abundance thresholds. The x-axis shows different abundance thresholds, and the y-axis shows different microbial taxa at species level. The color indicates the proportion of samples that contain each taxon at different abundance thresholds. (**D**) Relationship between the number and prevalence of taxa at different abundance thresholds. The lines show the prevalence of taxa, and the colors indicate different abundance thresholds. (**E**) Distribution of two species within Staphylococcus genus in H120, NCBI gut, and NCBI oral samples. Each point represents an OTU, and the x-axis shows the abundance of the OTU in the sample, and the y-axis shows the frequency of the OTU in the sample.

We further evaluated the MultiTax-human database using an independent cohort (Fig. 4). After quality control and denoising, we used the MultiTax-human database to assign taxa and identified 19 new taxa not present in the MultiTax-human database (Fig. 4A). We annotated the OTUs identified in the H120 data set with MultiTax-human, GTDB, GreenGenes2, Silva, and RDP. A comparison of the number of unclassified sequences in different databases at various classification levels (Fig. 4B) revealed that the MultiTax-human database had the smallest unidentified microorganisms, indicating its comprehensive coverage.

Further analysis of the core taxa in the H120 data set revealed a higher consistency in H120 gut samples compared with NCBI gut samples (Fig. 4D; Fig. S1 and S2); there were 204 strict core taxa in H120 samples. Notably, the OTUs with the highest occurrence rate was *Staphylococcus*, which also appeared in the gut and oral data from the NCBI public database (Fig. 4C and E). Leveraging the MultiTax-human database, we have further discerned potential novel *Staphylococcus* species, and the prevalence rate of these new taxa in oral and gut was relatively low.

### The MultiTax-human database web interface

**Fig 5:**
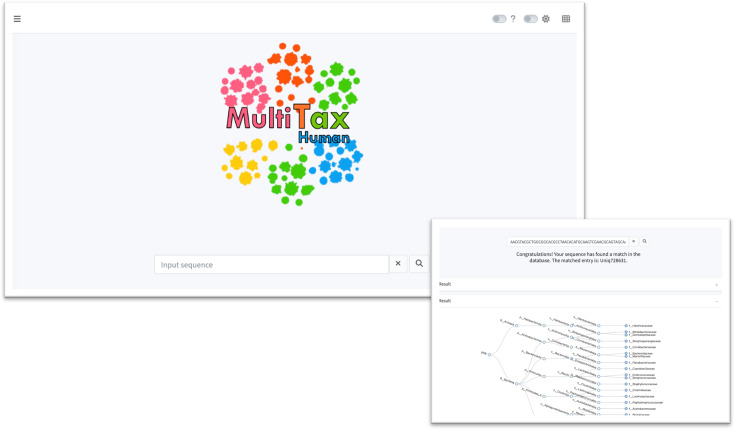
The web interface of the MultiTax-human database. We implemented a web application for the MultiTax-human database using Shiny framework, where users can submit a query sequence and obtain its taxonomic assignment in the MultiTax-human database and the corresponding annotations in other reference databases.

To enhance usability and accessibility, we developed a web application for the MultiTax-human database using the Shiny framework (Fig. 5). This application allows users to submit query sequences and retrieve taxonomic assignments from the MultiTax-human database, along with corresponding annotations from other reference databases. The code used to build the web application is available at https://github.com/zwbao/MultiTax-database. With the code, users can set up the platform locally and explore this high-resolution resource for human microbiome research. Additionally, our online platform will be available soon, further facilitating access for researchers.

## DISCUSSION

The creation of an extensive and updated reference database, such as MultiTax-human, is essential for advancing microbiome research and its applications in health studies. In this study, we developed the MultiTax-human database along with the MultiTax pipeline, an automated annotation system built on the GTDB framework. This resource provides high-resolution taxonomic classifications and extensive related data, making it a valuable tool for conducting detailed investigations into the human microbiome and facilitating further research in this field.

Focusing on 16S rRNA sequences in this study is strategic, considering their widespread use in microbiome studies due to their high taxonomic resolution and cost-effectiveness. While metagenomics offers a broader view of the microbial community, 16S sequencing remains a valuable tool, especially in clinical settings where quick, reliable, and cost-effective methods are paramount (21). The extensive coverage of high-quality full-length 16S rRNA sequences in the MultiTax-human database makes it an invaluable resource for detailed profiling of the human microbiome. Moreover, insights gained from 16S-based microbiome studies lay a foundational understanding that benefits and complements metagenomic approaches, making our work beneficial for researchers interested in both fields (22).

The implementation of the MultiTax pipeline, which re-annotates sequences from other databases based on GTDB, is a new approach that can accurately address the issue of inaccuracy and inconsistence among different databases. By adopting the global alignment algorithm, the MultiTax pipeline is able to effectively curate the consensus taxonomy from multiple databases, thereby enhancing the precision of microbial identification. It also increases the chance of identifying novel taxa, as demonstrated by the discovery of 339 new species in this study. This approach of incorporating multiple databases and deriving a consensus taxonomy effectively leverages the strengths of each individual database, while mitigating their weaknesses.

The MultiTax-human database encompasses 842,649 high-quality full-length 16S rRNA sequences from human-related samples. The extensive coverage of the database offers an unparalleled resource for the detailed profiling of the human microbiome. This study demonstrates the utility of the MultiTax-human database in identifying novel taxa and profiling the microbiota across various human body sites. The significant intersection between oral and gut microbiomes revealed in our study underscores the potential for future research into the interaction between these microbiomes and their role in health and diseases. Furthermore, the evaluation of the MultiTax-human database using an independent H120 cohort robustly substantiates the database’s comprehensiveness. And the high occurrence rate of the OTU Staphylococcus in both intestinal and oral data echoes previous findings of the widespread distribution of Staphylococcus in the human microbiome (23, 24). Given its extensive collection of human-related 16S sequences and its significant clinical implications, the MultiTax-human database emerges as an instrumental asset for clinical microbiome research, holding the promise to profoundly influence this evolving field.

Understanding the core microbiota is crucial for appreciating the stability and dynamics of the human microbiome (25). Our results demonstrate the importance of core microorganisms in maintaining the total microbial abundance, despite their low proportion in the total taxonomy, aligns with previous studies that have highlighted the critical role of core microbiota in maintaining the functional stability of the microbiome (26). The correlation between the core microbiota in breast milk and oral samples is particularly intriguing, hinting at potential shared microbial pathways and microbial seeding during early life (27).

The MultiTax-human database and MultiTax pipeline collectively represent significant advancements in 16S rRNA sequencing analysis. They provide an extensive and detailed reference for human microbiome studies, enhancing our ability to identify and annotate microbial taxa accurately. However, there are potential limitations and directions for further improvement. Our approach of re-annotating sequences from other databases based on GTDB may inadvertently bias the taxonomic classification toward GTDB. Furthermore, while the MultiTax-human database serves as an extensive repository for human microbiome research, it may not be suitable for non-human microbiome studies. Future iterations of the MultiTax pipeline could consider the inclusion of additional databases to further enhance the robustness of the consensus taxonomy.

In conclusion, the MultiTax-human database and pipeline provide a high-resolution resource for advancing microbiome research, offering improved taxonomic resolution for human-specific microbiome studies. Their ability to identify novel taxa and understand the core microbiota lays the groundwork for in-depth examination of the intricate relationship between the human microbiome and health. By offering a high-resolution view of the human microbiome, these tools can provide the foundations for designing targeted microbiome interventions, developing microbiome-based diagnostics, and elucidating the microbiome’s role in disease pathogenesis.

## Data Availability

The MultiTax reference database and MultiTax-human database are available for download via Google Drive. The download link can be found in the README file of the associated GitHub repository (https://github.com/zwbao/MultiTax-database). The code for constructing the MultiTax-human database and the MultiTax pipeline is available at https://github.com/zwbao/MultiTax-database.

## SUPPLEMENTAL MATERIAL

Figure S1 spectrum.01312-24-s0001.tiff Inter-individual divergence in NCBI Gut samples and H120 samples.

Figure S2 spectrum.01312-24-s0002.tiff Comparison of general core microbiota between NCBI Gut samples and H120 samples.

Table S1 spectrum.01312-24-s0003.xlsx Full-length 16S rRNA sequencing metadata of 1798 human source samples.

ASM does not own the copyrights to Supplemental Material that may be linked to, or accessed through, an article. The authors have granted ASM a non-exclusive, world-wide license to publish the Supplemental Material files. Please contact the corresponding author directly for reuse.
