# Supplementary figures and images for: MultiTax-human: an extensive and high-resolution human-related full-length 16S rRNA reference database and taxonomy
Source: Microbiol Spectr. 2025 Jan 16;13(2):e01312-24. doi: 10.1128/spectrum.01312-24 (PMC11792508; doi:10.1128/spectrum.01312-24)

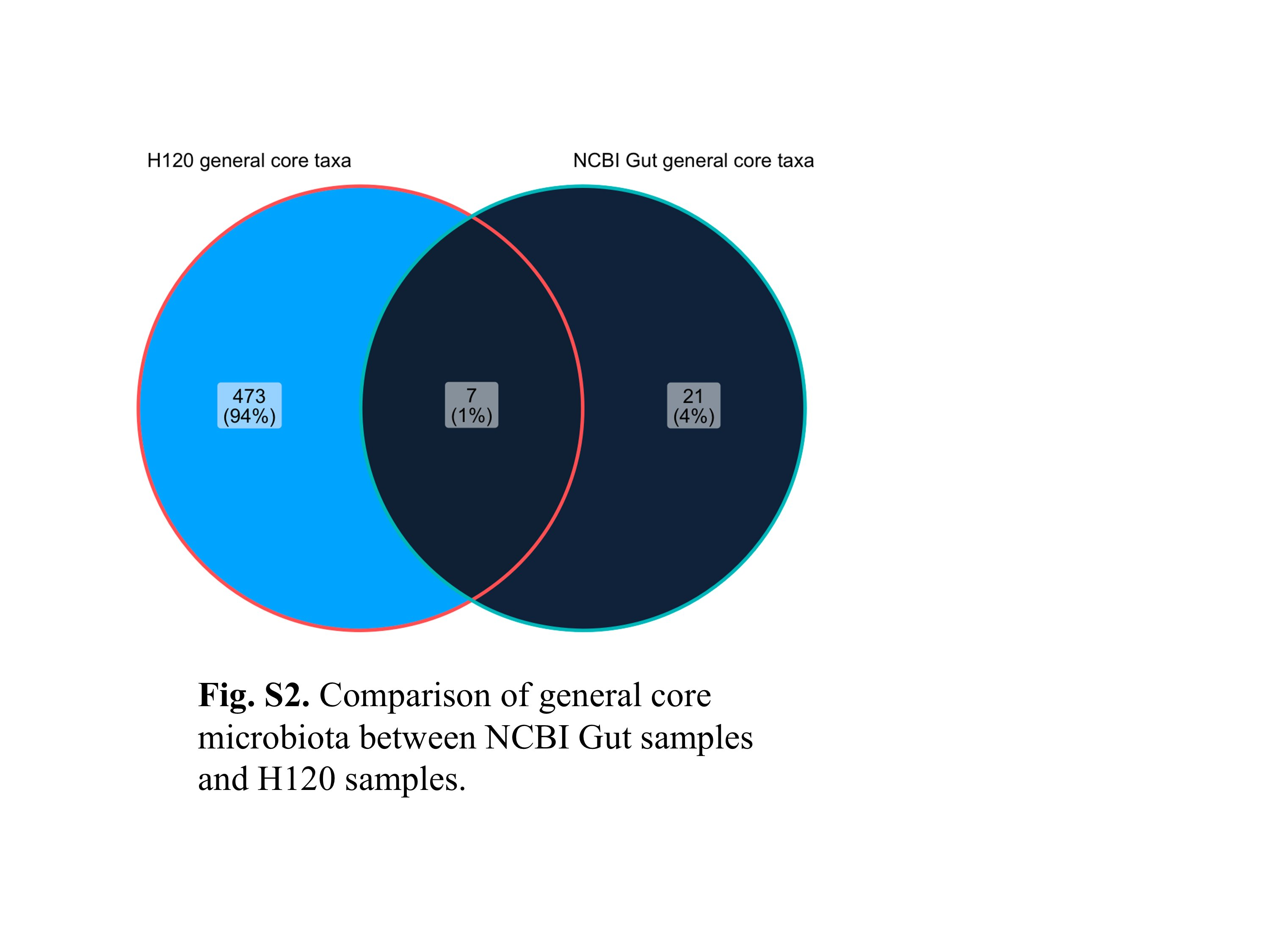

Supplement: Figure S2 — Comparison of general core microbiota between NCBI Gut samples and H120 samples. [file spectrum.01312-24-s0002.tiff]
